# Galleria mellonella as a versatile model for investigating Candida glabrata virulence and antifungal resistance

**DOI:** 10.1099/jmm.0.002127

**Published:** 2026-02-18

**Authors:** Charlie J. D. Holt, Catrin C. Wiliams, Jane Usher

**Affiliations:** 1 Medical Research Council Centre for Medical Mycology, University of Exeter, Exeter, UK; 2 School of Biosciences, University of Exeter, Exeter, UK

**Keywords:** *Candida glabrata*, *Galleria mellonella*, infection

## Abstract

*Candida glabrata* is an opportunistic fungal pathogen and a leading cause of invasive candidiasis, particularly in immunocompromised patients, where treatment is increasingly compromised by intrinsic and acquired antifungal resistance. Despite lacking morphological plasticity, *C. glabrata* employs distinct virulence strategies, including adhesin-mediated host colonization, intracellular survival within phagocytes, stress tolerance, iron acquisition and biofilm formation. This review synthesizes current knowledge of *C. glabrata* virulence and antifungal resistance mechanisms, with a particular focus on azole and echinocandin resistance driven by efflux pump regulation and *FKS* mutations. We critically evaluate the greater wax moth larva, *Galleria mellonella*, as a non-mammalian *in vivo* model for studying *C. glabrata* pathogenesis, host–pathogen interactions and antifungal efficacy. Evidence demonstrating concordance between *G. mellonella*, murine models and clinical outcomes is discussed, alongside the model’s limitations, including the absence of adaptive immunity. Collectively, this review highlights *G. mellonella* as a robust, cost-effective platform for dissecting *C. glabrata* virulence traits and for preclinical screening of antifungal therapies, supporting its growing role in translational fungal research.

## Introduction

Fungal infections are a significant global health concern, causing over 1.5 million deaths annually [1]. Among fungal pathogens, *Candida* species are responsible for a substantial proportion of invasive fungal infections, with *Candida albicans*, *Candida glabrata (Nakaseomyces glabratus*), *Candida krusei*, *Candida parapsilosis* and *Candida tropicalis* accounting for more than 90% of all candidiasis cases [2]. While *C. albicans* remains the most common causative agent of candidiasis, *C. glabrata* has emerged as a major opportunistic pathogen, particularly in immunocompromised patients [3]. Over the past decade, the prevalence of *C. glabrata* infections has doubled, largely due to its intrinsic and high-level acquired azole resistance [4] alongside its ability to rapidly acquire resistance to echinocandins, which are the current first-line therapy for invasive candidiasis.

Unlike *C. albicans*, a polymorphic fungus capable of morphological switches between yeast, pseudohyphal and hyphal morphologies as a virulence mechanism during infection [5], *C. glabrata* exists predominantly as a haploid yeast. Despite its lack of hyphal formation, *C. glabrata* exhibits several virulence traits that enable it to colonize host tissues, evade immune responses and establish infections [6,7]. These include adhesion to host surfaces via adhesin proteins, biofilm formation, secretion of hydrolytic enzymes, iron acquisition mechanisms and resistance to oxidative stress. Additionally, *C. glabrata* is particularly adept at surviving within macrophages, allowing it to persist in the host and contribute to recurrent or chronic infections [8].

The increasing clinical significance of *C. glabrata* infections necessitates the development of alternative treatment strategies and a deeper understanding of its pathogenic mechanisms. Despite its growing clinical relevance, *C. glabrata* remains far less studied than *C. albicans*, largely due to their fundamentally different biological strategies. Whereas *C. albicans* displays highly virulent, filamentation-driven pathogenesis and extensive host interaction data, *C. glabrata* is a nonfilamentous yeast whose virulence relies instead on stress tolerance, adhesion, immune evasion and intracellular persistence. These distinct traits present experimental challenges, as many established virulence readouts for *C. albicans* do not directly translate to *C. glabrata*. Consequently, there is a relative scarcity of mechanistic and comparative studies, particularly in alternative infection models. One of the key challenges in studying fungal virulence is the reliance on mammalian models, which are often costly and ethically complex and require extensive regulatory approval. As a result, there has been growing interest in using alternative *in vivo* models, including the greater wax moth larva, *Galleria mellonella*, to study *Candida* pathogenesis and antifungal drug efficacy. *G. mellonella* offers a low-cost, ethically acceptable model with innate immune parallels to mammals, enabling its growing use in *Candida* research [9].

**Fig. 1.**
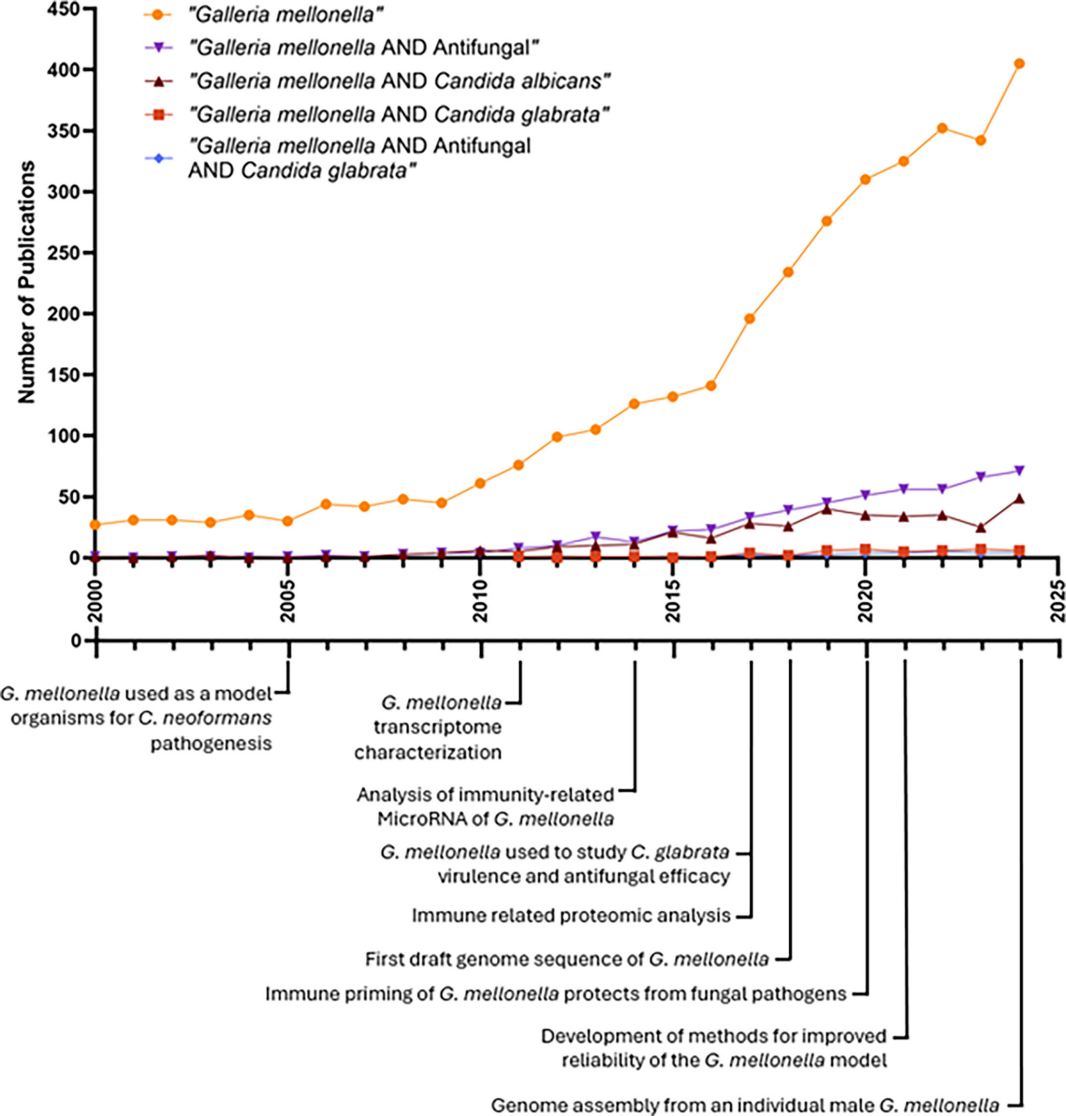
A timeline of *G. mellonella* publications with milestones of *G. mellonella* research. The model organism *G. mellonella* is increasingly used in research with increasing search results associated with keyword searches within the PubMed database. Keyword searches include ‘*Galleria mellonella*’ (orange circles), ‘*Galleria mellonella* AND Antifungal’ (purple inverted triangles), ‘*Galleria mellonella* AND *Candida albicans*’ (brown triangles), ‘*Galleria mellonella* AND *C. glabrata*’ (red squares) and ‘*Galleria mellonella* AND Antifungal AND *C. glabrata*’ (blue diamonds). The increasing number of publications depicted highlights the rapid expansion of *G. mellonella* research and underscores its growing importance as an infection model. The steady rise in studies using *G. mellonella,* particularly those focused on antifungal testing and *Candida* species, reflects both the model’s practical accessibility and its recognized translational value. Notably, the accelerated growth in publications over the past decade suggests heightened interest in nonmammalian systems for early-stage virulence and drug efficacy studies, driven by the need for cost-effective, ethically favourable and high-throughput alternatives to vertebrate models. Together, these trends demonstrate that *G. mellonella* is becoming increasingly embedded within the fungal research landscape, reinforcing its relevance and motivating continued methodological refinement and standardization. Created using GraphPad Prism 10.3.1. Key milestones in *G. mellonella* research were annotated using [9,73,80].

Over the past two decades, *G. mellonella* have been extensively used to investigate the virulence of various *Candida* species (Fig. 1). Notably, infection models using *G. mellonella* have demonstrated that *C. albicans* is highly virulent, with rapid filamentation and tissue invasion leading to high mortality rates in larvae [5]. In contrast, *C. glabrata* exhibits lower virulence in this model, with infections often resulting in prolonged survival of the larvae. However, recent studies suggest that *C. glabrata* virulence in *G. mellonella* can be enhanced under certain conditions, including co-infections with *C. albicans* or the acquisition of antifungal resistance mutations [10]. This highlights the utility of *G. mellonella* as a dynamic model for studying fungal interactions, adaptation and drug resistance.

Beyond virulence studies, *G. mellonella* has proven to be an effective platform for evaluating antifungal treatments. Larval models can be used to screen antifungal drugs at physiologically relevant concentrations, and the survival, fungal burden and immune response of larvae can be monitored over time [9,11]. Importantly, studies using *G. mellonella* have shown strong concordance with mammalian models and clinical outcomes [9,11, 12], further validating its relevance as a preclinical screening tool. Given the increasing burden of drug-resistant fungal infections, *G. mellonella* provides a valuable alternative for rapidly assessing the efficacy of new antifungal compounds before progressing to more complex mammalian studies.

Despite its advantages, there are some limitations to using *G. mellonella* in fungal infection research. Unlike mammalian models, *G. mellonella* lacks an adaptive immune system, meaning that long-term host–pathogen interactions and immune memory responses cannot be studied [13]. Additionally, while the larvae provide a useful approximation of fungal virulence, they do not fully replicate the complex interactions between fungal pathogens and mammalian tissues, particularly in the context of mucosal infections or bloodstream dissemination [14]. Nonetheless, the benefits of using *G. mellonella* including its low cost, high throughput and ethical advantages make it an indispensable tool for *Candida* research.

## Virulence mechanisms of *C. glabrata*

A key feature of *C. glabrata* pathogenicity is its ability to adhere to host surfaces, mediated by the epithelial adhesin (EPA) family [15]. Genes such as *EPA1, EPA6* and *EPA7* are upregulated during infection, promoting colonization of mucosal tissues and medical devices [16,17]. Adhesin expression is highly dynamic, responding to pH, nutrient status and oxidative stress, allowing persistence across diverse host environments [18]. To evade host immunity, *C. glabrata* modifies its cell wall to mask *β*-glucans from Dectin-1 and Dectin-2 recognition, reducing macrophage activation [16,19, 20]. Unlike *C. albicans*, it often survives intracellularly within macrophages, tolerating oxidative stress via antioxidant enzymes (e.g. catalase and superoxide dismutase) and nitric oxide (NO) detoxification by flavohaemoglobin (*YHB1*) [8,21]. This adaptation enables persistence and dissemination. Further, *C. glabrata* exhibits stress tolerance and replicative ageing traits, with older cells, specifically referring to replicatively aged mother cells, which accumulate bud scars over successive division. These cells exhibit enhanced survival under host-derived stresses and antifungal exposure compared with younger daughter cells, as opposed to stationary-phase or chronologically aged populations [22,23].

Iron acquisition also underpins virulence: high-affinity uptake systems (*FRE, FET3/FTR1*), haem utilization (*HMX1*) and storage proteins ensure growth in iron-limited environments such as the bloodstream [24]. Finally, biofilm formation enhances persistence on host tissues and medical devices. Biofilm-associated cells exhibit elevated efflux pump expression (e.g. *CDR1* and *PDH1*) and extracellular matrix (ECM) production, conferring substantial antifungal resistance [25].

## Antifungal resistance in *C. glabrata*

**Fig. 2.**
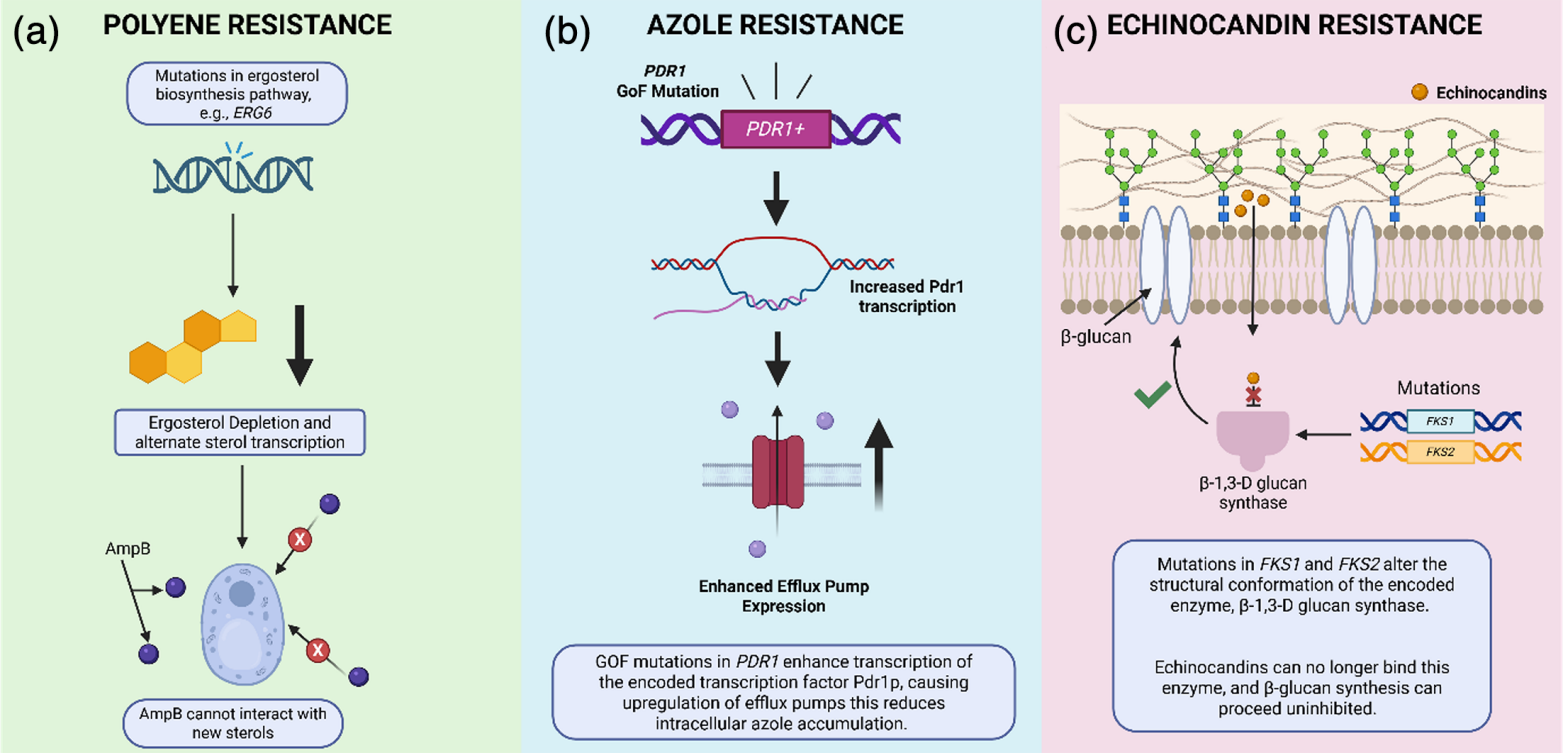
An overview of the main mechanisms of antifungal resistance in *C. glabrata*. The left-hand side panel (**a**) displays the main mechanism of polyene resistance in *C. glabrata,* where mutations in genes involved in the ergosterol biosynthesis pathway result in ergosterol depletion and increase transcription of alternate sterols which polyenes such as AmpB cannot bind, resulting in resistance. The middle panel (**b**) depicts the main mechanism of azole resistance in *C. glabrata*. Gain-of-function (GoF) mutations in *PDR1* (*PDR1^+^*) result in enhanced transcription of its encoded transcription factor Pdr1p, resulting in downstream upregulation of genes which encode efflux pumps which subsequently reduce intracellular azole accumulation. The right-hand side panel (**c**) describes the main mechanism of echinocandin resistance in *C. glabrata*. Mutations in the genes *FKS1* and/or *FKS2* result in changes in the structural confirmation of the encoded enzyme *β*-1,3-glucan synthase, meaning echinocandins can no longer bind and the enzyme can continue to participate in *β*-glucan synthesis [27,30,81, 82].

*C. glabrata* poses major therapeutic challenges due to its low-level intrinsic azole susceptibility, its ability to rapidly acquire high-level azole resistance and increasing echinocandin resistance. Currently, there are three classes of antifungals to which resistance in *C. glabrata* is increasingly documented [26] (Fig. 2). Azole resistance arises from overexpression of efflux pumps (e.g., *CDR1* and *CDR2*) due to gain-of-function mutations in the gene encoding the transcriptional regulator *PDR1* (*PDR1^+^*), and occasionally from *ERG11* mutations [27,28]. Echinocandin resistance is driven by hotspot mutations in *FKS1/FKS2*, reducing drug binding to *β*-1,3-glucan synthase [29,30]. Multidrug-resistant (MDR) *C. glabrata* isolates have been reported, in which strains carrying PDR1+alleles show cross-resistance to both azoles and echinocandins, further limiting available treatment options [30].

Clinically, these mechanisms limit azole use and threaten echinocandin efficacy, with resistant isolates often requiring amphotericin B treatment or combination therapy. The emergence of MDR strains underscores the urgent need for new antifungals and stewardship strategies.

Recent surveillance data indicate that antifungal resistance in *C. glabrata* continues to rise at a clinically significant pace. A 2024 population-based genomic study of 82 serial isolates reported *in vivo* acquisition of fluconazole or echinocandin resistance in one-third of patients, with multiple cases showing the emergence of resistant strains after initially susceptible isolates, demonstrating rapid adaptive evolution under drug exposure [26]. A 2025 multi-centre study from eastern China found fluconazole resistance rates of 21.7% among bloodstream isolates, along with emerging resistance to all three echinocandins in certain strains [31]. Global reviews further highlight that multidrug resistance, particularly combined azole and echinocandin resistance, is increasingly reported in *C. glabrata* and is rising more quickly than in most other *Candida* species, with the exception of *Candida auris* [32]. Together, these findings underscore that *C. glabrata* is one of the fastest-evolving *Candida* species in terms of antifungal resistance, driven by its strong capacity for rapid genomic adaptation.

## *G. mellonella* as a model for *Candida* research

The use of alternative animal models in fungal pathogenesis research has gained significant attention due to ethical concerns, high costs and the complexity of mammalian models. *G. mellonella* has emerged as a valuable model for studying fungal infections, including those caused by *Candida* species. This insect model offers several advantages, including a functional innate immune system, ease of handling and the ability to study host–pathogen interactions at a relatively low cost. *G. mellonella* has been extensively used to investigate the virulence mechanisms and antifungal resistance of *C. glabrata*, providing insights into adhesion, immune evasion, stress tolerance, biofilm formation and antifungal resistance mechanisms as evidenced thus far.

## Advantages of *G. mellonella* in *Candida* research

One of the main reasons for using *G. mellonella* in *Candida* research is its functional innate immune system, which shares similarities with mammalian immunity. The insect’s immune response is mediated by haemocytes, which function analogously to mammalian macrophages and neutrophils [33,35]. These haemocytes perform phagocytosis, generate reactive oxygen species (ROS) and release antimicrobial peptides (AMPs) in response to fungal infections, providing a useful platform for studying host–pathogen interactions [36,38]. Additionally, *G. mellonella* larvae can be maintained at a range of temperatures, including 37 °C, which is physiologically relevant for studying fungal infections in humans [39].

Compared to traditional mammalian models, *G. mellonella* is cost-effective, requires no ethical approval and allows for high-throughput screening of fungal virulence and antifungal drug efficacy [9]. The ability to inject standardized inocula (commonly 10 µl) directly into the larval haemocoel enables researchers to assess fungal burden, survival rates and immune responses in a reproducible manner [40,41]. Moreover, the transparent cuticle of the larvae allows for *in vivo* imaging of fungal progression and host responses, further enhancing its utility in *Candida* research [42,43]. Despite its suitability for high-throughput experimentation, the *G. mellonella* model also presents several practical challenges that must be carefully managed to ensure reproducibility. Establishing and maintaining a stable, disease-free larval colony requires an initial investment of time and resources, and larvae sourced from different suppliers or even different batches can vary substantially in size, developmental stage and baseline immune activity. Such batch-to-batch variability, along with sensitivities to temperature shifts, handling stress and inconsistencies in larval storage conditions, can introduce experimental noise and affect infection outcomes. These factors highlight the importance of rigorous standardization of larval selection, handling and inoculation procedures when employing *G. mellonella* in high-throughput studies.

## Assessment of *C. glabrata* virulence in *G. mellonella*

*C. glabrata* is an opportunistic fungal pathogen known for its ability to adhere to host surfaces, evade immune detection and resist antifungal drugs. *G. mellonella* has been instrumental in evaluating these virulence traits in a physiologically relevant setting. Studies have demonstrated that *C. glabrata* strains with mutations in adhesion-related genes, such as *EPA1*, exhibit reduced virulence in *G. mellonella*, confirming the importance of the epithelial adhesin (EPA) family in host colonization [16,18]. The upregulation of *EPA6* and *EPA7* during infection in *G. mellonella* further supports their role in fungal adherence and persistence within the host [18]. Immune evasion is another key virulence factor of *C. glabrata*, allowing the fungus to survive and proliferate within the host [8]. In *G. mellonella*, *C. glabrata* can modulate the immune response by altering its cell wall composition [36]. Previous work has shown that strains with reduced *β*-glucan exposure evade recognition by haemocytes, leading to decreased phagocytosis and a higher survival rate of fungal cells within the larvae [20]. This finding mirrors observations in mammalian models, where *C. glabrata* modifies its cell wall to avoid recognition by Dectin-1 receptors on macrophages [19].

## Biofilm formation and antifungal resistance in *G. mellonella*

Biofilm formation is a major contributor to *C. glabrata* pathogenicity, particularly in catheter-associated infections and medical device-related candidiasis. *G. mellonella* provides a valuable model for studying biofilm-associated infections, as fungal biofilms can be established within the larvae and analysed *in vivo*. Recent work demonstrated that *C. glabrata* biofilms formed in *G. mellonella* exhibit increased resistance to antifungal agents, similar to those observed in clinical settings [25]. The ECM produced during biofilm formation contributes to drug resistance by limiting antifungal penetration and enhancing efflux pump activity. Mutations in genes involved in ergosterol biosynthesis, such as *ERG11*, have been linked to increased azole resistance in *C. glabrata* biofilms, which can be effectively studied in *G. mellonella* [44].

Echinocandin resistance, driven by mutations in *FKS1* and *FKS2*, can also be assessed using *G. mellonella* [45]. In *C. glabrata*, echinocandin resistance is most commonly associated with well-defined hotspot mutations in FKS1 and FKS2, particularly within hotspot 1 (HS1) and hotspot 2 (HS2). Frequently observed mutations include FKS2 S663P, S663F, F659V, F659L and FKS1 S629P, all of which reduce echinocandin binding affinity to β-1,3-glucan synthase. Notably, FKS2 mutations are more prevalent and often confer higher levels of resistance than FKS1 alterations in *C. glabrata*, reflecting the species’ reliance on FKS2 as the dominant glucan synthase under stress conditions. While analogous hotspot regions exist in *C. albicans* and *C. auris*, the specific amino acid substitutions differ considerably, making the mutational landscape of *C. glabrata* more tightly concentrated around HS1 substitutions such as S663P. These species-specific patterns highlight both the conserved structure of the glucan synthase complex and the unique regulatory pathways, shaping echinocandin resistance in *C. glabrata*. Larvae infected with echinocandin-resistant *C. glabrata* strains exhibit higher survival rates in the presence of caspofungin, indicating that echinocandin resistance leads to persistent infections despite antifungal treatment. These findings are consistent with clinical observations of echinocandin treatment failure in patients with invasive *C. glabrata* infections [29].

## Oxidative stress and iron acquisition mechanisms in *G. mellonella*

Oxidative and nitrosative stress tolerance is crucial for *C. glabrata* survival within the host [21,23, 46]. The ability to detoxify ROS and NO produced by immune cells allows the fungus to evade immune clearance [21,23]. *G. mellonella* has been used to study the oxidative stress response of *C. glabrata*, revealing the upregulation of antioxidant enzymes such as catalase (*CTA1*) and superoxide dismutase (*SOD1*) in infected larvae [46]. The flavohaemoglobin *YHB1*, which detoxifies NO, has also been shown to contribute to fungal survival in *G. mellonella*, supporting its role in immune evasion [47].

Iron acquisition is another essential factor for *C. glabrata* pathogenesis, as iron is a critical micronutrient for fungal growth [24]. *G. mellonella* has been used to study the expression of iron uptake genes, such as those encoding ferric reductases (*FRE* family) and the iron transport system (*FET3/FTR1*).

## Application of *G. mellonella* in antifungal drug testing

Given its advantages as a fungal infection model, *G. mellonella* has been widely used for antifungal drug screening [9]. In a typical infection assay, larvae are inoculated by injection of a defined fungal suspension directly into the haemocoel, most commonly via the last left proleg, using a standardized injection volume of ~10 µl [40,41]. For *C. glabrata*, inoculum sizes generally range from 10^5^ to 10^7^ yeast cells per larva, allowing dose-dependent assessment of virulence, drug efficacy and host survival [9]. Following infection, larvae are incubated at 30 or 37 °C, with the latter commonly used to reflect human physiological conditions.

Antifungal compounds can be administered either prophylactically (pre-infection) or therapeutically (post-infection), depending on the experimental objective. Therapeutic regimens are more frequently employed and typically involve antifungal administration within 1–2 h post-inoculation, although delayed treatment schedules have also been used to model established infection and treatment failure. Drugs are delivered by haemocoelic injection at concentrations selected to approximate clinically relevant plasma levels, and treatment efficacy is assessed using survival curves, fungal burden measurements and host immune readouts [9,11].

Studies have demonstrated that antifungal susceptibility profiles observed in *G. mellonella* closely resemble those reported in mammalian models and clinical isolates, supporting its utility as a predictive *in vivo* screening platform [9]. The emergence of MDR *C. glabrata* strains has intensified the need for alternative therapeutic strategies, and *G. mellonella* has been effectively used to evaluate both monotherapy and combination regimens. These include echinocandins in combination with amphotericin B, as well as novel antifungal agents such as ibrexafungerp, a glucan synthase inhibitor [48,50]. Notably, ibrexafungerp has demonstrated efficacy against echinocandin-resistant *C. glabrata* strains in larval infection models, supporting its potential as a therapeutic alternative [51].

Overall, the *G. mellonella* model has proven to be a versatile and informative system for studying *C. glabrata* pathogenesis, virulence mechanisms, biofilm-associated infections, antifungal resistance, oxidative stress responses and iron acquisition strategies. Its functional innate immune system, low cost, experimental flexibility and ability to recapitulate key aspects of mammalian infection make it an attractive alternative to traditional vertebrate models. As antifungal resistance continues to rise, the integration of *G. mellonella* into antifungal drug development pipelines will remain instrumental in accelerating the identification and optimization of new therapeutic strategies against *C. glabrata*.

## Future outlooks and clinical relevance of *G. mellonella* in *Candida* research

### Cost-effective and ethical alternative to mammalian models

A key advantage of the *G. mellonella* system is its markedly lower cost relative to mammalian infection models. Commercially sourced *G. mellonella* larvae typically cost £0.20–£0.50 per larva, with no specialized housing requirements and no need for regulated animal facilities. In contrast, the purchase cost of a single laboratory mouse is generally £20–£40, with daily housing costs of £1–£3 per animal, alongside additional expenses associated with personal licensing, ethical approval, procedural training and regulated containment facilities. A standard infection experiment involving 100 animals would therefore cost approximately £20–£50 using *G. mellonella*, compared to £3,000–£6,000 for a comparable murine study when accounting for animal purchase, housing and regulatory overheads. This substantial cost difference allows for larger cohort sizes, increased experimental replication and higher-throughput antifungal screening in *G. mellonella*, making it an accessible model for early-stage pathogenesis and drug efficacy studies.

Mammalian models, particularly murine models, are the gold standard in fungal research due to their ability to mimic human pathophysiology (Table 1). However, these models require specialized housing, ethical approvals and substantial financial investment, limiting their widespread use. In contrast, *G. mellonella* offers a more accessible alternative, allowing researchers to perform large-scale studies at a fraction of the cost. The larvae can be maintained at a range of temperatures, including 37 °C, which is essential for mimicking human fungal infections [9,52,55]. Moreover, the infection process is straightforward, with standardized inoculation techniques enabling reproducible results across different experimental settings [9].

**Table 1.** Comparative overview of key biological, immunological, pharmacological and transcriptomic features of the *G. mellonella* model versus murine/human systems

Aspect	*G. mellonella*	Murine/human
Phagocytes	Haemocytes (neutrophil-like) (lectin-mediated phagocytosis, ROS production, extracellular traps)	Macrophages, neutrophils (lectin-mediated phagocytosis, ROS production, extracellular traps)
Humoral response	AMPs (gallerimycin and galiomycin), melanin cascade (phenoloxidase)	AMPs, complement, cytokines
Virulence factors (*EPA* gene family, Dtr1)	Active, temperature-dependent, studied	Similar but more complex interactions
Ageing effects in *C. glabrata*	Older cells resist phagocytosis and killing	Also, resistance, selection via neutrophils
Adaptive immunity	None	Present
Model correlation	Good predictors of murine trends	Gold standard, with immunological depth

## *G. mellonella* immune system and its relation to murine and human immunity

*G. mellonella* only possesses innate immune systems and lacks adaptive immune responses. Innate immunity within *G. mellonella* is divided into cellular immunity (i.e. haemocyte response) and humoral immunity (i.e. AMPs, lysozymes and melanization) (Table 1) [36,56,58]. There are six haemocyte classes within *G. mellonella* which display functional similarity to mammalian phagocytes; notably, granular cells and plasmatocytes perform phagocytosis, nodulation, encapsulation and clotting [35,37, 59, 60]. Multiple studies have shown that insects possess numerous pattern recognition receptors (PRRs) which are analogous to mammalian PRRs, such as lectins which are functionally reminiscent of C-type lectin receptors [61]. Notably, *G. mellonella* display dose-dependent immune responses to *β*-glucan inoculation, where administration of high *β*-glucan doses resulted in increased haemocyte density and elevated expression of AMPs [61]. Additionally, treatment of larvae with caspofungin and micafungin elicits immunomodulatory effects where enhanced haemocyte density is observed mimicking observations in murine studies [62,63].

Due to the similarities observed between larval and murine innate immune systems, *G. mellonella* represents a good model for initial screening of immune responses to *C. glabrata* infections. However, haemocytes in *G. mellonella* have been described as more functionally akin to mammalian neutrophils than macrophages [33,34, 59, 64]. Haemocytes, like neutrophils, are circulatory cells which are capable of nodulation, encapsulation and formation of extracellular traps [59,64], whereas macrophages are antigen-presenting tissue-resident cells which can secrete cytokines [65]. Within humans and murine models, *C. glabrata* infection predominantly elicits internalization by macrophages, and its ability to survive and replicate within macrophages is a key factor underpinning its virulence [8]. This coupled with the lack of an adaptive immune system limits the utility of *G. mellonella* models in characterizing the immune response to *C. glabrata* infection.

## *C. glabrata –G. mellonella* interactions

*G. mellonella* models can be used to accurately characterize virulence of *C. glabrata* isolates, with dose-dependent responses observed [9]. Notably, isolates presented significantly increased virulence when larvae were incubated at 37 °C relative to incubation at 30 °C, highlighting the role of temperature-dependent expression of many *C. glabrata* virulence genes [9]. A key virulence factor, Dtr1, a multidrug transporter, is dramatically up-regulated inside haemocytes even 100-fold after 24–48 h and contributes to survival against oxidative and acidic stress during phagocytosis; deleting Dtr1 reduces larval killing by ~30% [66]. Furthermore, age-related differences in *C. glabrata* affect interactions: older cells are more resistant to hydrogen peroxide and neutrophil killing and show lower phagocytosis rates by both *Galleria* haemocytes and human neutrophils; they also exhibit remodelled cell walls and increased fluconazole resistance, leading to higher virulence in larvae [22].

## Correlation between *Galleria* and murine models

Virulence trends observed in *G. mellonella* often correlate with murine systemic infection models, validating the insect as a surrogate for screening virulence or antifungal efficacy. However, murine models remain the gold standard, given richer immune complexity, costlier but more clinically relevant.

Table 1 summarizes major similarities and distinctions relevant to infection biology and antifungal research. Alongside innate immune parallels including haemocyte-mediated phagocytosis, ROS production and melanization, the table highlights constraints such as the absence of adaptive immunity in *G. mellonella*. Pharmacological comparability is supported by evidence showing that antifungal susceptibility patterns and treatment responses in *Galleria* frequently mirror those observed in murine models and clinical isolates, including concordance for azoles and echinocandins. Transcriptomic studies further indicate that fungal gene expression profiles during *Galleria* infection reflect those seen in mammalian systems, underscoring the model’s translational relevance for studying stress responses, metabolic adaptation and virulence-associated pathways.

In conclusion, *G. mellonella* provide a valuable, inexpensive *in vivo* model for studying *C. glabrata* virulence. *G. mellonella* models can be used to study *C. glabrata*–phagocyte interactions, such as Dtr1-mediated stress resistance and age-related virulence, with studies largely reflecting murine and human dynamics. Yet, key distinctions remain: haemocytes lack adaptive immune features, complement is replaced by melanization and cytokine signalling is absent. Therefore, while *Galleria* captures crucial innate immune-fungal dynamics, murine models remain essential for comprehensive virulence profiling.

## Future perspectives

*G. mellonella* provide a promising platform for investigating antifungal resistance mechanisms and screening novel treatment strategies. One area of active research involves the integration of transcriptomic and proteomic analyses with *G. mellonella* infection models. These omics-based approaches can reveal key regulatory pathways involved in *C. glabrata* pathogenesis, providing valuable targets for future drug development. Recent transcriptomic studies have demonstrated that gene expression profiles of *C. albicans* during *G. mellonella* infection closely mirror those observed in mammalian models, further validating the translational relevance of this system [67,68]. Similar analyses for *C. glabrata* are likely to provide deeper insights into its adaptive responses to antifungal treatment and host immune pressures.

*C. glabrata* possesses a highly plastic genome which allows for large-scale genomic changes within isolates which can facilitate resistance to antifungals [4,11, 26, 69,71]. These genetic changes are often unstable, allowing for rapid adaptation to environmental stresses encountered within the host, with bloodstream isolates of *C. glabrata* from the same patient possessing distinct karyotypes [72]. Future studies should interrogate whether *G. mellonella* models can be used to investigate adaptation of *C. glabrata* isolates to antifungal drugs within larvae and whether recovered colonies from haemolymph extractions display karyotypic differences as observed in murine models and human infections [40].

*G. mellonella* has become a valuable, tractable model for studying *C. glabrata* virulence, immunity and antifungal response, offering low cost, high-throughput experimentation and translational relevance that often aligns with murine and clinical outcomes. Yet important gaps remain, including limited understanding of how *C. glabrata*’s nonfilamentous physiology, stress-adapted survival strategies and rapid genomic plasticity shape host interactions, as well as incomplete characterization of key immune processes such as haemocyte-mediated melanization and extracellular trap formation. Progress is also hindered by inconsistent methodologies across laboratories, underscoring the need for standardized protocols. Future priorities include integrating multiomics approaches to map host–pathogen dynamics, using the model to track within-host evolution of antifungal resistance and expanding comparative studies across *Candida* species. Together, these efforts will strengthen the utility of *G. mellonella* and advance mechanistic insight into *C. glabrata* pathobiology and therapeutic vulnerabilities.

## Abbreviations

AMPs: antimicrobial peptides
ECM: extracellular matrix
HS1: hotspot 1
MDR: multidrug-resistant
NO: nitric oxide
PRRs: pattern recognition receptors
ROS: reactive oxygen species
